# Segregation of information about emotional arousal and valence in horse whinnies

**DOI:** 10.1038/srep09989

**Published:** 2015-04-21

**Authors:** Elodie F. Briefer, Anne-Laure Maigrot, Roi Mandel, Sabrina Briefer Freymond, Iris Bachmann, Edna Hillmann

**Affiliations:** 1 ETH Zürich, Institute of Agricultural Sciences, Universitätstrasse 2, 8092 Zürich, Switzerland; 2 Agroscope - Swiss National Stud Farm, Les Longs Prés, P.O. Box 191, 1580 Avenches, Switzerland; 3 Koret School of Veterinary Medicine, Robert H. Smith Faculty of Agriculture, Food and Environment, the Hebrew University, Rehovot 76100, Israel

## Abstract

Studying vocal correlates of emotions is important to provide a better understanding of the evolution of emotion expression through cross-species comparisons. Emotions are composed of two main dimensions: emotional arousal (calm versus excited) and valence (negative versus positive). These two dimensions could be encoded in different vocal parameters (segregation of information) or in the same parameters, inducing a trade-off between cues indicating emotional arousal and valence. We investigated these two hypotheses in horses. We placed horses in five situations eliciting several arousal levels and positive as well as negative valence. Physiological and behavioral measures collected during the tests suggested the presence of different underlying emotions. First, using detailed vocal analyses, we discovered that all whinnies contained two fundamental frequencies (“F0” and “G0”), which were not harmonically related, suggesting biphonation. Second, we found that F0 and the energy spectrum encoded arousal, while G0 and whinny duration encoded valence. Our results show that cues to emotional arousal and valence are segregated in different, relatively independent parameters of horse whinnies. Most of the emotion-related changes to vocalizations that we observed are similar to those observed in humans and other species, suggesting that vocal expression of emotions has been conserved throughout evolution.

Expression of emotions and perception of emotional states play an important role in social species. Indeed, emotion expression informs individuals about the probable intention of behaviors of others and therefore, regulates social interactions^1^. Vocal expression of emotions has been extensively studied in humans (“affective prosody”^2^,^3^). However, human voice also depends on socio-cultural and linguistic conventions that can act as confounding factors in the study of affective prosody^4^. In most non-human animals, vocalizations are assumed to be under lower voluntary control than in humans. Animal vocalizations should thus reflect emotions more directly than human voice^5^, and could serve as valuable models for studies on human affective prosody. They could also serve as ideal non-invasive tools to assess emotions in animals, in which the subjective, conscious component of emotions cannot be accessed^6^.

A promising approach to study animal emotions is through their two main dimensions (“dimensional approach”^7^); 1) emotional arousal (excitation, e.g. calm versus excited) and 2) emotional valence (negative or positive, e.g. sad versus happy)^8^. Vocal correlates of emotional arousal have been relatively well studied (see reviews^6^,^9^). Vocalizations usually become longer, louder, and are produced at faster rates, with higher and more variable frequencies when arousal increases^6^. By contrast, there is considerably less knowledge on vocal indicators of valence (i.e. differentiating between negative and positive state)^6^. Several types of vocalizations have been shown to indicate either positive or negative emotions^10^,^11^,^12^. However, changes in vocal parameters according to emotional valence have rarely been investigated^6^. Another limitation of the research on vocal expression of emotions in animals is that very few studies investigated vocal correlates of both emotional arousal and valence in the same species (but see^13^,^14^,^15^). In addition, the emotional situations that have been used often differ in both their valence and their arousal, and the effects of these two dimensions on vocal parameters are not tested independently. Therefore, more studies are needed to investigate how emotions are encoded in vocalizations, and to advance our understanding of the phylogenetic continuity of vocal correlates of emotional arousal and valence between humans and other animals.

We hypothesized that, in order to be effectively encoded within vocalizations, cues to emotional arousal and valence could be segregated in different acoustic features or in temporally distinct vocal segments (segregations of information^16^). Segregation of information such as individual identity and group membership, body weight or size and condition, social status and hormonal state, exists in the vocalizations of several species (e.g. rock hyrax, *Procavia capensis*^17^; banded mangoose, *Mungos mungo*^18^). Alternatively, arousal and valence could be encoded in the same parameters, in which case a trade-off would exist between the two dimensions, resulting in one dimension being more accurately communicated than the other (e.g. identity and quality in fallow deer, *Dama dama,* sexually selected vocalizations^19^).

To test whether the encoding of the two emotional dimensions is in accordance with the segregations of information hypothesis^16^, or if they are encoded in the same parameters (trade-off hypothesis^19^), we investigated vocal correlates of emotional arousal and valence in domestic horses, *Equus caballus*. Horses are very social animals that, in the wild, live in harems (stallion, females and foals) or in bachelor bands (young or old stallions without a harem)^20^. Vocal expression of emotions should benefit horses by regulating social interactions within groups. Horse vocalizations have been rarely investigated. This species produces several types of calls; whinnies, nickers, squeals, blows, snores, snorts, roars, and groans^21^,^22^. Squeals have been shown to contain information about dominance rank^23^, and whinnies about sex, body size and individuality^24^. A recent study showed that the overall structure of whinnies differs between negative and positive situations^25^. However, this study did not specifically test which vocal parameters are affected or not by valence.

We combined new frameworks recently adapted from human to animal research to analyze vocalizations (source-filter theory^26^) and emotions^8^. We placed horses in one control situation and four social situations, triggering various levels of emotional arousal and different valence. Heart rate is a well-established indicator of physiological stress^27^, which is linked to emotional arousal during situations associated with both positive and negative valence^28^. We thus measured heart rate to determine the arousal of our situations, and assumed that variations in heart rate were linked to underlying emotions triggered by our situations (i.e. indicative of emotional arousal)^29^. In the absence of well-established valence indicators, we inferred the valence of our situations from knowledge of the function of emotions and on horse behavior. We focused our analyses on whinnies, which are produced in both separation (negative valence) and greeting (positive valence) contexts to maintain or regain contact with affiliates or offspring^21^,^22^. We tested the hypothesis that the emotional arousal and valence experienced by horses are indicated by particular vocal profiles. Our vocal analyses systematically revealed two fundamental frequencies in all whinnies, suggesting biphonation, a rare phenomenon among mammals that had not been described in previous studies in horse vocalisations^21^,^23^,^24^. We thus first ruled out potential alternative explanations to biphonation, before testing the effect of emotional arousal and valence on vocal parameters, including those related to the two fundamental frequencies. Additionally, in order to confirm underlying emotions, we tested if the resulting emotional arousal levels, as well as the presupposed valence of the situations, were accompanied by physiological and behavioral changes measured during the tests^30^,^31^. We defined the parameters that changed according to increased arousal levels as reliable cues to arousal. Similarly, we defined the parameters that changed consistently from negative to positive valence as reliable cues to valence^29^.

## Results and discussion

We tested 20 privately owned horses of various breeds and age, housed in five different farms (3–5 horses per farm; Table 1). We designed four situations potentially eliciting different levels of emotional arousal and characterized by negative or positive valence, which were likely to trigger whinnies. These situations involved separation (supposedly of negative valence^20^) and reunion (supposedly of positive valence^32^) with either all group members (supposedly high emotional arousal) or only one group member (supposedly moderate emotional arousal). In the negative situation “All-Leave”, all the other horses from the farm (2–4 horses depending on the farms; hereafter “group members”) were removed, while the subject was kept in its home box or paddock alone. In the positive situation “All-Return”, all the group members were brought back towards the subject following the All-Leave situation. The two other negative and positive situations were similar to the first ones, except that only one group member (the “Companion”, and not all group members) was taken away from the subject (“Companion-Leaves”) and then walked back towards it (“Companion-Returns”). The four situations were compared to a control situation (“Control”), during which the subject and all other group members were in their home box or paddock and were not manipulated.

**Figure 1:**
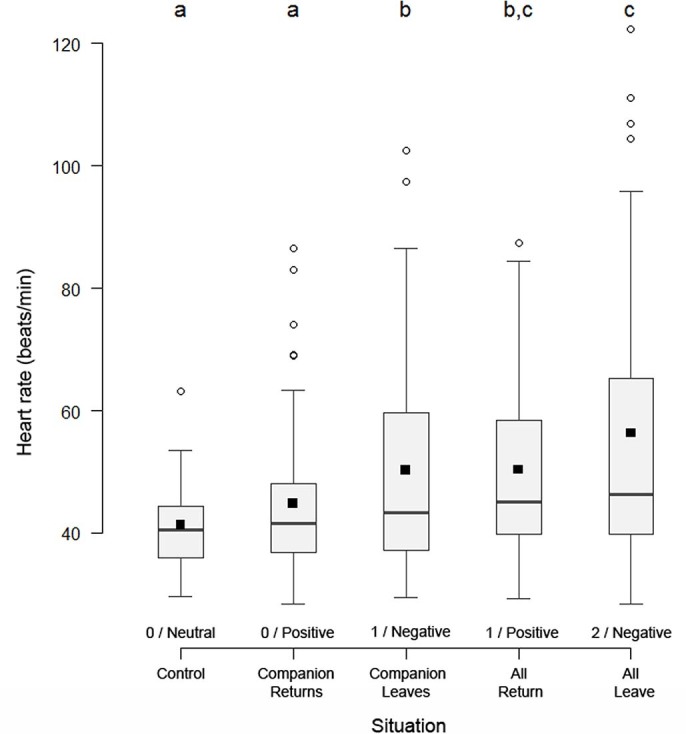
Heart rate as a function of the emotional situations. Heart rate (raw values) for each of the experimental situations; box plot: the horizontal line shows the median, the box extends from the lower to the upper quartile and the whiskers to 1.5 times the interquartile range above the upper quartile or below the lower quartile; circles indicate outliers. Same letters (a, b, c) indicate that situations did not significantly differ (linear mixed-effects models compared with likelihood-ratio tests; log transformed heart rate values controlled for sex, age and body weight of the horses, order of the situations (All or Companion tests first), day of experiment, individual and farm identity). Based on these results, situations marked with “a” received an emotional arousal level of 0, situations marked with “b” received an arousal level of 1, including All-Return situation, and the All-Leave situation (marked with “c”) was considered of arousal level of 2. Resulting emotional arousal levels (0–2) and valence (Negative, Neutral and Positive) corresponding to the situations are indicated below each box (arousal level / valence).

The actual emotional arousal level triggered by our situations was assessed from the horse heart rate, measured during the tests^29^. The analysis of heart rate as a function of emotional situations revealed three emotional arousal levels; 0 for Control and Companion-Returns, 1 for Companion-Leaves and All-Return, and 2 for All-Leave (Figure 1; heart rate, arousal level 0: 43.93 ± 11.58 beats/min; arousal level 1: 50.27 ± 16.96 beats/min; arousal level 2: 56.32 ± 23.46 beats/min; see Methods for more details and statistics).

The valence of the situations was inferred from knowledge of the function of emotions and of horse behavior. Positive emotions result from encounters with rewarding stimuli that enhance fitness. By contrast, negative emotions are triggered by punishing stimuli that threaten fitness^8^. Horses are highly social animals and separation from conspecifics is thus stressful for them^20^,^33^. This situation would, in the wild, potentially threaten fitness through greater exposure to predators. On the other hand, reunion with group members triggers greeting vocalizations between affiliated pairs of horses^21^,^22^, and this situation could enhance fitness by lessening exposure to predators. Furthermore, the small numbers of individuals per farm (3 to 5 horses) in our study ensured strong familiarity and potentially close bonds between horses. The valence of the situations was thus assumed to be negative for the situations involving group members leaving (All-Leave and Companion-Leaves), neutral for the Control situation (Control), and positive for the situations where group members were coming back (All-Return or Companion-Returns). We then verified that the arousal levels and valence of our situations were accompanied by consistent physiological and behavioral changes measured during the tests (see Supplementary Results).

### Vocal structure of whinnies

**Figure 2:**
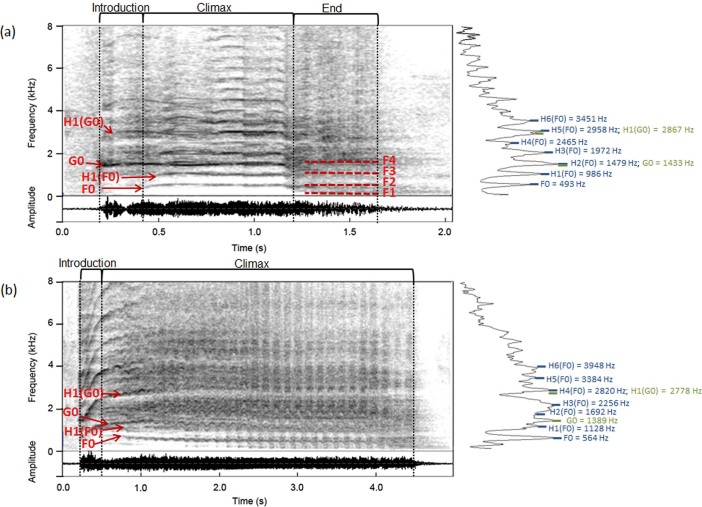
Spectrograms of two whinnies. (a) and (b) spectrograms (above), oscilliogams (below) and 100 Hz cepstral-smoothed spectra (right; frequency versus amplitude) of two whinnies produced by different horses. Whinny (a) contains the three typical parts; the introduction, the climax and the end, where putative formants (F1-F4) could be measured. Whinny (b) contains only the introduction and the climax, which is frequency modulated. The end part is not present and putative formants could not be measured in this kind of whinny. For both whinnies (a and b), F0 (lower fundamental frequency) and G0 (higher fundamental frequency) are indicated on the spectrograms and cepstral-smoothed spectra, as well as the harmonics (i.e. multiples) of F0 (H*n*(F0)) and of G0 (H*n*(G0)). As shown on the cepstral-smoothed spectra, the harmonics of F0 and G0 do not occur at the same frequencies. These whinnies are available as audio files (Audio S1).

Whinnies are the longest, loudest and most common horse vocalization. Three parts have previously been described; 1) the “introduction”, which is tonal and high in frequency; 2) the “climax”, which is a long, often frequency and amplitude modulated part; and 3) the “end”, which is low in frequency and amplitude, and composed of a pulse-train structure (Figure 2; Audio S1)^24^. According to the source-filter theory of speech production^34^, vocalizations are generated by vibrations of the vocal folds (source, determining the fundamental frequency), and are subsequently filtered by the supralaryngeal vocal tract (filter, producing amplified frequencies called “formants”^35^). In order to extract both source- and filter-related vocal parameters of whinnies, we assumed that vocal production in horses is fundamentally similar to humans and to other mammals on which this framework has been successfully applied^26^. In total, we extracted 19 source- and filter-related vocal parameters as well as intensity and duration measures (see Table 2 for abbreviations, and Supplementary Methods for analysis description) from 267 whinnies produced by 18 horses (2 horses did not produce whinnies; Table 1).

Previous studies on whinnies only measured the fundamental frequency in the highly tonal introduction part (easily identifiable fundamental frequency), and argued that this parameter is not always identifiable in the climax and end parts^24^. However, after carrying out detailed analyses, we noticed that the climax part is always (i.e. 100% of whinnies) composed of a second, lower fundamental frequency and its corresponding harmonics (Figure 2; Audio S1), suggesting biphonation (i.e. presence of two independent fundamental frequencies that are not integer multiples^36^). As biphonation is a rare phenomenon in mammals and had not been previously described in horses, we first carried out a detailed vocal analysis to rule out alternative explanations to biphonation, before testing the effect of emotional arousal and valence on the structure of whinnies. We measured the two fundamental frequencies throughout the introduction and climax parts. These frequencies are not clearly visible in the end part of the whinnies, which is characterized by a more chaotic-like pulse-train structure, with pulses potentially corresponding to the vibrations of the vocal folds^37^, suggesting a drop in fundamental frequency (Figure 2). The lower fundamental frequency (399.22 ± 99.39 Hz, range = 52–1050 Hz, *n* = 267 whinnies), starting at the beginning of the climax, is hereafter referred as “F0”. We referred to the higher fundamental frequency (1543.26 ± 326.45 Hz, range = 493–3012 Hz, *n* = 260 whinnies), starting at the beginning of the whinny, as “G0” (Figure 2)^36^.

**Figure 3:**
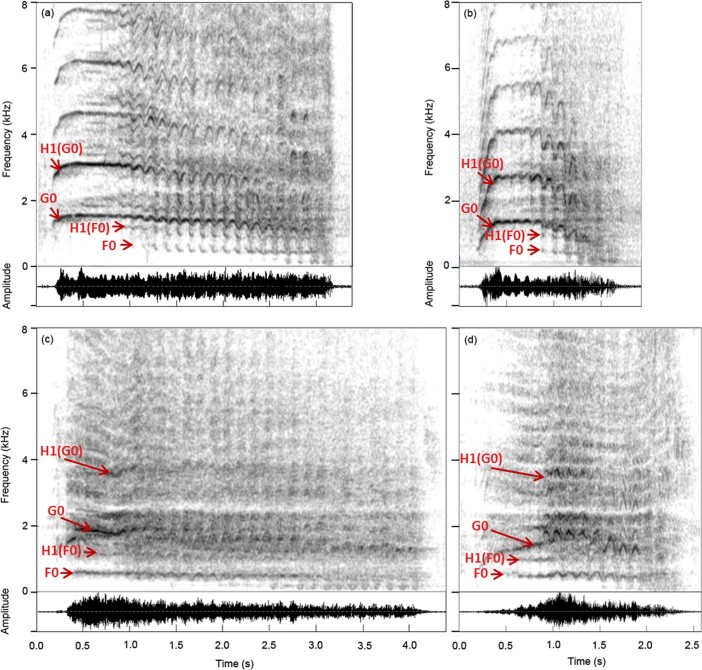
Negative and positive whinnies. (a) and (c) spectrograms (above) and oscilliogams (below) of whinnies produced during the negative situations by two different horses; (b) and (d) spectrograms (above) and oscilliogams (below) of whinnies produced during the positive situations by the same two horses (different horses than for Figure 2). F0 (lower fundamental frequency) and G0 (higher fundamental frequency) are indicated, as well as the first harmonics (i.e. multiples) of F0 (H1(F0)) and of G0 (H1(G0)). Positive whinnies (b) and (d) are shorter in duration and have a lower G0 (start and mean) than negative whinnies (a) and (c). These whinnies are available as audio files (Audio S2).

In order to rule out alternative explanations to biphonation, we first verified that F0 does not simply result from a register transition (i.e. abrupt change in fundamental frequency)^38^,^39^. In this case, G0 and F0 should not overlap. However, in horse whinnies, G0, which starts at the beginning of the whinny, can be observed and measured throughout the introduction and climax, even after F0 appears at the beginning of the climax (Figures 2 and 3). G0 and F0 thus overlap over 79.40 ± 20.52% of the call on average ((Dur-DurIntro/Dur); *n* = 267 whinnies; Table S1). Secondly, we verified that F0 and G0 are not harmonically related (i.e. not integer multiples of each other). For instance, F0 could be a sub-harmonic of G0 that appears following a bifurcation (i.e. change in regime from normal phonation, where vocal folds are synchronized, to sub-harmonic regime, where one vocal fold is moving faster than the other and having twice or more the period of the other^40^). If they were harmonically related, G0 and F0 should have been positively correlated with each other both between horses (i.e. be the product of one another; G0 = n * F0), as well as over time within each whinny (i.e. have the same frequency over time (“contour”); *r*^2^ between G0 and F0 contour approaching 1)^41^. G0 was 4.39 ± 2.24 times higher than F0 on average (*n* = 260 whinnies). The average G0mean and F0mean were not correlated between horses (Spearman's rank correlation: *r*^2^ = 0.002, *p* = 0.88, *n* = 18 horses). This suggests that horses with a higher G0 do not necessarily have a higher F0 and vice versa. G0 and F0 contours measured in 71 whinnies, in which we had been able to extract these contours throughout the entire introduction and climax, were significantly correlated for 63 of them (positively correlated in 56 whinnies and negatively correlated in 7 whinnies). When the correlation was significant (*p* < 0.034), *r*^2^ ranged from 0.06 to 0.92 (Spearman's rank correlation: *r*^2^ = 0.51 ± 0.23, *n* = 63 whinnies). F0 and G0 are thus neither the product of one another, not fully correlated over time (*r*^2^ close to 1), indicating that they are not harmonically related.

Next, we ruled out various spectrogram artifacts (aliasing, clipping and reverberation), which could be mistaken for biphonation or other non-linear phenomena (e.g. subharmonics and deterministic chaos^36^). Aliasing occurs when the vocalization contains frequency components above one half of the sampling frequency. These components appear as image frequencies in the spectrogram and can be confounded for biphonation. In our case, this artifact can be ruled out as our sampling frequency was 44100 Hz, which is more than twice the highest frequencies observed in horse whinny (mean highest frequency measured on a spectrum = 13677 ± 2947, range = 9153–21157 Hz, *n* = 2 whinnies per horse, 18 horses (36 whinnies in total)). Clipping results from severe overloading and produces abrupt changes in the spectrogram, which may resemble bifurcations. Yet, we controlled for overloading by adjusting the recording level during our recordings, and by excluding from our analyses whinnies that were overloaded (see oscillograms in Figures 2 and 3). Finally, reverberation is due to the resonance properties of the environment and appears as an artificial sound prolongation^36^. This artifact can also be ruled out in our case, as the second frequency (F0) always appears at the beginning of the climax part, and not at the end of the whinny or when two whinnies overlap.

In addition to the previously mentioned spectrogram artifacts, sidebands (or side frequencies) that are non-harmonically related to the fundamental frequency can appear on the spectrogram symmetrically on each side of the fundamental and its harmonics, as a result of strong and rapid amplitude modulations^42^,^43^. However, G0 and F0 could still clearly be observed in whinnies after we modified them by artificially removing the amplitude modulation (Figure S1). Similarly, sidebands on each side of the fundamental frequency and its harmonics can appear in narrow-band spectrograms as a result of rapid frequency modulation^44^. Yet, in this case, such sidebands should always be apparent at equal distance above and below G0 and its harmonics. The absence of this clear pattern in most whinnies (Figures 2 and 3), as well as the lack of appearance of rapid frequency modulations when changing the bandwidth of spectrograms (narrow-band to broad-band)^44^, allowed us to also rule out this hypothesis. Therefore, there is strong evidence suggesting that F0 and G0 are not artifacts.

Our results show that F0 and G0 are not generated by artifacts, nor are they harmonically related, suggesting real biphonation in whinnies. G0 was present, in addition to F0, in all whinnies, indicating that the presence of two fundamental frequencies is a common feature of this type of call. Whinnies hence differ from the vocalizations of other mammalian species, in which biphonation, mostly found in young primates and canids, appears sporadically (e.g. 44% of the calls in dhole, *Cuon alpinus*^41^; 60% of the calls of African wild dog, *Lycaon pictus*^36^). The vocal production mechanisms responsible for biphonation in whinnies could be the following; involvement of vocal fold extensions, vortex-shedding at the glottal constriction, source-tract coupling or, more probably, asynchronous vibration pattern of the vocal folds^36^,^41^. Further detailed anatomical investigation of horse vocal production apparatus are required to understand the mode of production of F0 and G0.

### Vocal correlates of emotional arousal and valence

After describing the vocal structure of horse whinnies, we carried out linear mixed-effects models to test the effect of emotional arousal and valence on all the vocal parameters measured, including those related to F0 and G0. Subjects never whinnied during the Control situation (neutral valence), and the effect of valence on vocal parameters could thus only be assessed by comparing situations of negative and positive valence (Table 3). Because the arousal levels and valence attributed to the analyzed whinnies were correlated (Spearman's rank correlation: *r*^2^ = 0.47, p < 0.0001; *n* = 267 whinnies), the effects of the two emotional dimensions on vocal parameters were not tested in the same models^45^. Instead, we ran one first set of models with arousal level as an explanatory factor, and another second set with valence^29^.

Our analyses of vocal parameters as a function of the emotions triggered by the experimental situations revealed 12 parameters that were influenced by emotional arousal (Table 3; see Table S1 for raw values). Dur (duration), G0start, G0max, G0mean (G0-related parameters), F0start, F0max (F0-related parameters), Q24%, Q50% and Q75% (energy quartiles) all increased with arousal levels, while AMextend and AmpVar (amplitude-related parameters) decreased (see Table 2 for abbreviations and definitions). F0mean was also affected by arousal, but did not change consistently with increasing arousal levels (level 0 > 1 < 2). F4mean (fourth putative formant) tended to increase, but not significantly, with arousal. Ten parameters were influenced by valence (Table 3; see Table S1 for raw values). Dur, G0start, G0max, G0mean, F0max, Q24%, Q50% and Q75% all decreased from negative to positive valence, while AMextend and AmpVar increased. F0start and AMrate tended to decrease from negative to positive valence, but these effects were only marginally significant (0.05 < *p* < 0.06). The other parameters were neither affected by arousal, nor by valence (Table 3).

When a parameter was significantly affected by both emotional arousal and valence, we used a model selection procedure based on Akaike's information criterion (AIC), to identify which of arousal or valence best explained the variation in each parameter value^29^. We used AIC adjusted for small sample sizes (AIC_C_), because AIC_C_ converges to AIC as the sample size increases and should thus be used by default^45^. The model (including arousal or valence) with the lowest AIC_C_ can be considered as the best model^46^. This model selection procedure revealed that the variation in F0max, AmpVar, Q24%, Q50% and Q75% was better explained by arousal than valence (Table 4). Conversely, the variation in Dur, G0start, G0max, G0mean and AMextend was better explained by valence than arousal levels (Table 4). For F0max, AmpVar, G0max and AMextend, the difference between the AIC_C_ values of the two models (ΔAIC_C_) was lower than 3, indicating that the models including arousal and valence were competitive. The model including arousal had 78% and 62% chance to be the best model for F0Max and AmpVar, respectively. The model including valence had 81% and 73% chance to be the best model for G0max and AMextent, respectively. For Q25%, the ΔAIC_C _was lower than 4, indicating that the model including valence had less support by the data than the model including arousal, which had 88% chance to be the best model.

To summarize, according to our criteria, F0start, Q50% and Q75% were reliable cues to arousal, because they were changing consistently with arousal and were clearly more affected by arousal than valence (ΔAIC_C _> 7). By contrast, Dur, G0start and G0mean were clearly more affected by valence than arousal (ΔAIC_C _> 7) and were therefore reliable cues to valence. Similar analyses carried out on physiological and behavioral parameters measured during the tests showed that the emotional arousal and valence of our situations were reflected by physiological and behavioral changes in the horses, suggesting underlying emotions (see Supplementary Results).

In order to examine clustering among parameters, we then carried out a principal component analysis (PCA), including all the vocal parameters measured in five randomly selected whinnies per horses (*n* = 9 horses, i.e. horses that produced at least 5 whinnies in which all 19 parameters were successfully measured; Table 1). The PCA generated six principal components (PCs) that exceeded Kaiser's criterion (eigenvalues >1) and accounted for 83% of the variation in the original data set. Among the six vocal parameters selected as good cues to horse emotions, those indicating arousal (F0start, Q50%, Q75%) were clustered in PC1, while those indicating valence (G0start and G0mean) were clustered in PC2. Finally, Dur, which also indicated valence, loaded highly on PC6 (Table S2).

Our study revealed that whinnies produced during high emotional arousal situations have a higher F0 (F0start) and energy distribution (i.e. energy quartiles; Q50% and Q75%) than those produced during low arousal situations. These results could be explained by 1) an increase in sub-glottal pressure and/or vocal fold tension producing an increase in F0, and 2) an increase in pharyngeal constriction or a less pronounced retraction of the larynx, resulting in a higher energy distribution, with an increase in arousal levels^2^,^35^,^47^. A raise in F0 and a shift in energy distribution towards higher frequencies with increasing arousal have been commonly observed in humans^2^,^3^, as well as in other mammals (e.g. pig, *Sus scrofa*^48^; tree shrew, *Tupaia belangeri*^49^; squirrel monkey, *Saimiri sciureus*^13^; reviews^6^,^9^). In particular, among all the studies reviewed in Briefer^6^, and in which F0 was measured (21 studies), F0 consistently increased with arousal. F0 and the energy distribution seem to be reliable and consistent cues to arousal across mammals, and even in birds (zebra finch, *Taeniopygia guttata*^50^).

Whinnies produced during positive situations were shorter in duration (Dur) and had a lower G0 (G0start and G0max) than those produced during negative situations (Figure 3; Audio S2). The change in duration can be explained by shorter expirations, resulting in shorter whinny duration during positive situations, compared to negative ones. However, in order to explain valence-related changes to G0, we would need to find the source of production of this parameter, which requires further examination. A decrease in duration between negative and positive situations, as revealed in our study, was also found in dogs^51^, and squirrel monkey^13^. These results are in accordance with the motivation-structural rules^52^, which states that calls produced during appeasing contexts are generally of shorter durations than those produced during aggressive contexts^53^. A lower pitch (G0 in our case) in positive compared to negative situations was highlighted in squirrel monkey^13^, and African elephant (*Loxodonta africana*^15^)*.* This suggests that vocal correlates of emotional valence could have been, similarly as those of emotional arousal, conserved throughout evolution. Alternatively, vocal correlates of emotional arousal could have been highly conserved, while those of valence could be more species specific. This latter hypothesis is supported by the fact that none of the vocal cues to valence found in goats (*Capra hircus*), in which a similar experimental procedure was used^29^, are similar to those that we found in horses.

Our results suggest that vocal expression of emotions in horses is in accordance with the hypothesis of segregation of information about emotional arousal and valence in different vocal parameters. Indeed, F0start, Q50%, Q75% were found to be good cues to emotional arousal, while Dur, G0start and G0mean were found to be good cues to valence. A PCA including all 19 measured vocal parameters revealed a segregation of cues to emotional arousal (F0start, Q50%, Q75%) and valence (Dur, G0start and G0mean) in different PCs, suggesting their independence. Emotional arousal and valence are therefore encoded in different parameters, which seem to be relatively independent of each other.

We found that putative formant frequencies were affected by body weight (decrease in frequencies with weight increase; Figure S2 and Table S3). A similar negative correlation between body weight/size and formant frequencies has been found in most mammals studied to date, because of the strong dependency of vocal tract length on body size (e.g. goat^54^; koala, *Phascolarctos cinereus*^55^). However, apart from the mean frequency of the fourth putative formant (F4mean), which tended to change with emotional arousal, these frequencies measured in horse whinnies were neither affected by arousal nor by valence. This could be due to the fact that we were able to measure putative formants only when the end part of whinnies was present (53% of the whinnies of each horse on average; see Supplementary Methods). Therefore, our sample size was smaller for putative formant frequencies than for other vocal parameters and might not have been sufficient to detect a link with emotions.

## Conclusions

We discovered that horse whinnies were composed of two fundamental frequencies (F0 and G0), suggesting biphonation. F0 and the energy spectrum indicated emotional arousal, while G0 and whinny duration indicated emotional valence. The function of non-linear phenomena (i.e. biphonation, subharmonics and deterministic chaos^36^) is not clear, but these particularities could allow individuals to generate highly complex and unpredictable vocalizations^40^. Biphonation has been suggested to enhance, among others, identity cues^56^,^57^. Our results show that the presence of two fundamental frequencies can also function as a means of emotion expression, with each frequency encoding one emotional dimension (i.e. arousal and valence). As emotional arousal and valence are each encoded by vocal parameters that seem relatively independent of each other, vocal expression of emotions in horses corresponds more to the segregation of information hypothesis^16^ than the trade-off hypothesis^19^. This suggests that emotional arousal and valence can both be effectively and simultaneously communicated in this species. Vocal communication of emotions could allow horses to regulate social interactions within groups. Further playback experiments could test if conspecifics perceive these emotional-related changes to the acoustic structure of whinnies^24^,^58^. Our approach allowed us to identify clearly which parameters were mostly influenced by each emotional dimension. We believe that this approach will lead to better knowledge of vocal correlates of emotions in animals, which could help to understand the phylogenetic continuity of emotion expression between animals and humans, through cross-species comparisons^29^.

## Methods

### Subjects and management conditions

Twenty horses were tested in May and June 2013 (Table 1). All the horses had been in their respective farm for at least 6 months. For each tested horse, we identified the group member that, according to the farm owner, elicited the highest number of vocalizations during separation (“Companion”). During the night, the horses were housed in single boxes (*n* = 5 horses) or in boxes with paddocks either individually (*n* = 10 horses) or in groups of two or three horses (*n* = 5 horses). During the day, they were kept outdoors, either individually in adjacent fields (*n* = 6 horses) or in groups of two to four horses (*n* = 14 horses).

### Experimental procedure

For each farm, the experiment was conducted on four successive days. The first day (“habituation day”) consisted of a habituation to the physiological measurement equipment (heart-rate monitor; see below *Response measures* for details) and to the situations, in order to minimize stress linked to novelty. Each of the second, third and fourth day (“test days”) consisted of a repetition of the four emotional situations (All/Companion*Leave/Return). This repetition allowed us to increase the sample size of whinnies produced during the situations.

The procedure was similar for the four days (i.e. habituation day and three test days). The horses were tested one by one, as follows; we placed the heart-rate monitor on the subject and waited five minutes (habituation day) or two minutes (test days) for the horse to get used to it. Following this time period, the “Leave” situation started (i.e. All-Leave or Companion-Leave). Two to four experimenters (depending on the number of horses at the farm) took all the other horses (“All tests”) or only the companion (“Companion tests”; in a counterbalanced order between and within horses; i.e. All tests followed by Companion tests or Companion tests followed by All tests) on a halter and walked away from tested horse's home box/paddock. The direction in which the experimenters walked away with the group member(s) changed between days to lessen the potential habituation. When out of sight of the tested the horse, the experimenters waited one minute (habituation day and first test day), two minutes (second test day) or five minutes (third test day) before coming back. This increase in time out of sight across days reduced habituation to the situations, thus maintaining a high emotional arousal level throughout the three days of tests and increasing the probability of eliciting whinnies. After the time out of sight elapsed, the “Return” situation started (i.e. All- or Companion-Return). The experimenters walked back with the group member(s) towards the subject's home box/paddock and released it(them) in its(their) home box/paddock. Then, the subject was left unmanipulated for five minutes, which allowed it to resume normal activities and its heart rate to reach baseline values, as displayed on the physiological analysis software. After this interval, the second pair of negative and positive situations started, and only the companion or all the other horses were moved away from the tested horse, depending on which tests had been carried out first (All or Companion tests). The procedure for the second pair of situations (Leave and Return) was identical to the one described above.

During the situations, two experimenters were standing 5–10 m away from the subject to operate the camera, the sound recorder and the physiological analysis software. For horses kept in paddocks, the access to the box was prevented to allow the experimenters to easily film the behavior of the horse at all times. Finally, the control situation was carried out on one of the four days of the experiment (one or two horses per farm per day), before the emotional situations started. We equipped the subject with the heart-rate monitor and waited two minutes for the horse to go back to normal activities. The situation then lasted 4 min. It did not elicit any whinny. However, this situation allowed us to obtain baseline values for physiological and behavioral data.

### Determination of the emotional arousal of the situations

To determine emotional arousal levels, we tested for differences in heart rate between situations (Figure 1). The heart rate of the horses differed according to the situation (linear mixed-effects model (LMM): *X*^2^*_3_ = * 35.49, *p* < 0.0001). The lowest heart rate values were obtained for the Control situation. These values were not different from those obtained for the Companion-Returns situation (Control vs Companion-Returns; LMM: *X*^2^*_1_ = * 2.67, *p* = 0.10). Therefore, we attributed an arousal level of 0 (lowest) to these two situations. The heart rate values measured during the Companion-Returns situation were significantly lower than those measured during the Companion-Leaves situation (Companion-Returns vs Companion-Leaves; LMM: *X*^2^*_1_ = * 10.28, *p* = 0.001), which, in turn, were similar to the values measured during the All-Return situation (Companion-Leaves vs All-Return; LMM: *X*^2^*_1_ = * 1.59, *p* = 0.21). Companion-Leaves and All-Return situations were thus assigned an arousal level of 1 (intermediate). Finally, the values for the All-Leave situation were not significantly different from those obtained for the All-Return situation (arousal level = 1) after Bonferroni correction (All-Return vs All-Leave; LMM: *X*^2^*_1_ = * 4.58, *p* = 0.032; Bonferroni, α = 0.01). However, because the values for the All-Leave situation were significantly higher than those for the Companion-Leaves situation (arousal level = 1; Companion-Leaves vs All-Leave; LMM: *X*^2^*_1_ = * 11.08, *p* = 0.0009), we attributed an arousal level of 2 (highest) to the All-Leave situation.

### Response measures

Vocalizations were recorded during the tests at distances of 5–10 m from the vocalizing animal using a Sennheiser MKH-70 directional microphone, connected to a Marantz PMD-671 numeric recorder (sampling rate: 44.1 kHz). They were imported into a computer and saved in WAV format at 16-bit amplitude resolution. We used Praat v.5.3.41 DSP Package and Seewave^59^ for subsequent analyses. Calls were visualized on spectrograms in Praat (FFT method, window length = 0.03 s, time steps = 1000, frequency steps = 250, Gaussian window shape, dynamic range = 60 dB). They were classified as whinny, nicker, squeal or sigh according to their acoustic characteristics^21^,^22^. We analyzed all good quality whinnies (i.e. low level of background noise) that were separated by at least 10 s (total = 267 whinnies from 18 horses; 201 negative and 66 positive; 13.35 ± 11.39 whinnies per horse; range = 0 (2 horses) – 35 whinnies; Table 1), in order to prevent pseudoreplication^54^ (consecutive calls are more likely to be homogeneous). Whinnies produced when the group member(s) was(were) leaving or out of sight were considered as negative. Whinnies produced when the group member(s) was(were) returning and visible, and when the subject was facing its(their) direction with ears pointing forwards were considered as positive. We extracted all vocal parameters using a custom built program in Praat. This program batch processed the analyses and the exporting of output data^60^. In order to prevent biases linked to the settings used for the analyses, for each horse, the best settings to analyze the vocal parameters were entered in the script, and both negative and positive whinnies of the subject were analyzed^29^. In total, we included 19 source- and formant-related parameters in our analyses. As the mechanism of vocal production in horses is not known, we made the following assumptions in order to extract putative formants using Linear Predictive Coding analysis in Praat; that the source and filter are relatively independent, that the source has an approximately 6 dB/octave falling spectral slope, and that the vocal tract can be approximated as a straight uniform tube, whose length can be estimated from head length (from the tip of the muzzle to the middle of the posterior part of the mandible^61^; see Supplementary Methods). The vocal parameters measured are listed in Table 2 and detailed in the Supplementary Methods.

Physiological measures were collected using a wireless non-invasive monitor (MLE120X BioHarness Telemetry System, Zephyr), fixed to a surcingle placed around the subject's heart girth. ECG gel was applied on the electrodes before each use. The data (continuous ECG trace and breathing wave, i.e. inhalation/exhalation cycle) were transmitted and stored in real time to a laptop using LabChart software v.7.2 (ADInstrument) for later analyses. During the tests, one experimenter entered comments in the software indicating when the group member(s) was(were) leaving and returning. This allowed us to measure the following physiological parameters at the exact moment when these events occurred: heart rate, root mean square of successive inter-beat interval differences (RMSSD), respiration rate and skin temperature (Table S4). We analyzed these parameters, when possible (i.e. good quality signal, clearly visible heart beats on the ECG trace and inhalation/exhalation on the breathing wave), for up to 30 s during the Leave tests (starting when all the doors of the boxes/paddocks were closed and when the experimenters started to walk away with all group members or with only the companion) and Return tests (starting from the time when the group member(s) was(were) visible and when the subject was orientated in its(their) direction). This duration (30 s) corresponded to the minimum duration taken by the experimenters to lead the group member(s) out of sight of the subject or to return. During the control situation, parameters were analyzed over up to 1 min of good quality signal (total situation duration = 4 min).

For each selection, we ensured that the software tracked the heart beats (ECG trace) and the inspiration–exhalation cycles (breathing wave) correctly. Parts of the ECG trace when an artrio-ventricular block could be observed (i.e. usually while resting; one heart beat missing every 3–4 beats) where excluded^62^. Then, the heart rate (beats/min), respiration rate (breaths/s) and skin temperature (°C) averages were obtained automatically from the software. Individual inter-beat intervals (ms) were also extracted, in order to assess heart-rate variability by calculating RMSSD (ms). When the parameters had to be averaged over several selections inter-spaced by noisy recording, a weighed arithmetic mean was calculated based on the length of each segment. On average, for each horse and each test day, we obtained physiological data over 21.30 ± 7.87 s during the All-Leave situation, 21.80 ± 6.07 s during the All-Return situation, 23.60 ± 6.87 s during the Companion-Leaves situation, 22.05 ± 6.39 s during the Companion-Returns situation. We obtained physiological data over 55.37 ± 8.82 s during the Control situation (*n* = 20 horses; see Supplementary Results for the effect of emotions on physiological parameters other than heart rate).

The behavioral parameters were scored from the videos of the tests, filmed using a Canon Legria FS2000 camcorder. We scored behavioral parameters that had been performed by at least 50% of the horses (i.e. 10 horses) during the tests (listed in Table S4). They were scored using The Observer software XT v.11 (Noldus), similarly as the physiological parameters, for 30 s during the Leave and Return tests, and for 1 min during the Control situation. During these periods, behaviors were scored either as occurrence (for discrete behaviors; “Point Events”) or as duration (for behavior lasting in time; “State Events”). We then calculated the frequency of occurrence per minute for the Point Events (indicated in min^−1^ in Table S4), and the proportion of the total time spent performing the behavior for State Event. The analyses were carried out on these frequencies of occurrence or proportions (see Supplementary Results for the effect of emotions on behavioral parameters).

### Statistical analysis

We tested for differences in heart rate between situations using a linear mixed-effects model (lmer function, lme4 library) in R 3.0.2 software. This model included heart rate as a response variable, and the sex, age and body weight of the horse, as well as the order of the situations (i.e. if Companion tests or All tests were carried out first or second each day), as fixed factors to control for their effect. The situation (All-Leave, All-Return, Companion-Leaves, Companion-Returns and Control) was included as a fixed factor. Finally, horse identity nested within the farm was included as a random factor crossed with the date of the test, in order to control for repeated measurements of the same subjects, and for farm and day differences (e.g. differences linked to increased time spent out of sight throughout the three days of tests). Then, two-by-two comparisons between the emotional situations were carried out using linear mixed-effects models including the same fixed and random factors. We applied a Bonferroni correction at α = 0.01 (0.05/5 comparisons; Figure 1) for theses posthoc tests. Based on these results, we ranked the situations according to the heart-rate values they triggered; we attributed the highest arousal level to the situation triggering the highest heart rate and the lowest arousal level to the one triggering the lowest heart rate. Situations that did not differ in heart rate were considered to be of the same arousal level (Figure 1)^29^.

After establishing arousal levels for all of the emotional situations, we carried out further models to test the effect of arousal and valence on the other vocal, physiological, and behavioral parameters measured (Table 2 and S4 for a list of parameters). Each model included the parameter as a response variable (one model per parameter) and the same control and random factors as listed above (sex, age and body weight of the horse, as well as the order of the situations as fixed factors - see Table S3 for results of these control factors; identity of the horses nested within farm, and the date of the test, as crossed random factors). The proportion of time spent moving was also included as a fixed factor for the physiological parameters, to control for its effect. For models including skin temperature as a response variable, we additionally added the outdoor temperature for each test day as a fixed factor to control for its effect. We ran one first set of models with arousal level as a fixed effect and another set with valence as a fixed effect. Then, for each parameter that was significantly affected by both arousal and valence, we used a model selection procedure based on Akaike's information criterion adjusted for small sample sizes (AIC_C_) to identify which of arousal or valence best explained the variation in each parameter value^29^. The model with the lowest AIC_C_ is considered as the best model^46^. When the difference between the AIC_C_ values of two models (ΔAIC_C_) is less than 2 units, both models have support and can be considered competitive. Models with ΔAIC_C_ ranging from 3 to 7 have considerably less support by the data, models with ΔAIC_C_ > 10 are poorly supported, and ΔAIC_C_ > 20 have no empirical support. Akaike weights (*ωi*) indicate the probability that a particular model has more or less support from the data among those included in the candidate models^46^.

The residuals were checked graphically for normal distribution and homoscedasticity. To satisfy assumptions, we used a log transformation for Q75%, AMextend and DurIntro, HR, RMSSD, RespRate (see Table 2 and S4 for abbreviations). Some of the behavioral parameters measured in proportions were logit-transformed (EarsForward, EarsHorizontal, EarsAsymetric and LookForward). These log- or logit-transformed physiological, vocal and behavioral parameters were then input into linear mixed-effects models fit with Gaussian family distribution and identity link function (lmer function, lme4 library) in R 3.0.2 software. The remaining behavioral parameters (see Table S4) did not meet statistical assumptions despite logit transformation. They were thus transformed to binomial data (behavior occurs = 1 or does not occur = 0; apart for Locomotion (%) and HeadMov (min^−1^): >10 = 0 and ≤ 10 = 1), and input into generalized linear mixed models fit with binomial family distribution and logit link function (glmer function, lme4 library) in R 3.0.2 software. For each model, we assessed the statistical significance of the factors by comparing the model with and without the factor included using likelihood-ratio tests (LRT). The LRT statistics follows a *χ*^2^-distribution with degrees of freedom equal to the difference in the number of parameters. To compare models with LRT and with AIC_C_, all models were fit with maximum likelihood estimation (see Supplementary Results for the effect of emotions on physiological and behavioral parameters).

To test if the vocal parameters determined as good cues to emotional arousal and valence were independent from each other, we used a PCA. This analysis allowed us to examine clustering among parameters within the generated principal components. As PCA requires a random sample from the population, with each individual contributing equally^63^, we considered only horses that had produced at least 5 whinnies in which the 19 parameters (Table 2) had been successfully measured (*n* = 9 horses). For horses with more of such whinnies, we selected five of them pseudo-randomly, in a way that would maximize the number of calls produced under each emotional arousal level and valence (e.g. 1–2 whinnies for each arousal level 0–2; total = 45 whinnies).

Three horses were not tested with all of the Remove-All situations because they elicited stress levels that were too high (see below in *Ethical Note*). Due to problems encountered with measuring devices, one horse could not be filmed entirely during the Companion tests on one of the days. Therefore, sample sizes differ between parameters (see sample sizes in Table 3 and S5). All statistical analyses were performed with in R 3.0.2 software. The significance level was set at α = 0.05. All means are given with standard deviations.

### Ethical note

All experiments were carried out in accordance with the current laws of Switzerland. This study was approved by the Swiss Federal Veterinary Office (approval number VD 2689). The negative situations lasted not more than 5 min each (i.e. from the time the group member(s) leave(s) and until it(they) return(s)). Furthermore, we used situations that are likely to be experienced by horses in their everyday life (e.g. when one of the horse in the group is being ridden). In case of high levels of stress experienced by a horse during one of the situation on the habituation or test days, as shown by its heart rate as well as by the amount of whinnies produced and the duration of locomotion (e.g. constantly calling and moving), it was not tested anymore during the following days with this situation. This happened for two horses with the Remove-All situation during the habituation. These horses were thus tested with the Companion tests only. Another horse was too stressed by the Remove-All situation during the last test days. Therefore, the test was aborted and behavioral and physiological data from this test were discarded.

## Author Contributions

EFB designed the study, collected the data, analyzed the vocal parameters, carried out the statistics and wrote the paper. A-LM helped to design the study, collected the data, analyzed the behavioral parameters and commented on the paper. RM helped to design the study, analyzed the physiological parameters and helped to write the paper. SBF designed the study, participated in the data collection, and commented on the paper. IB helped to design the study and commented on the paper. EH helped to design the study and commented on the paper. All authors read and approved the final manuscript.

## Supplementary Material

Supplementary Information Supplementary information

Supplementary Information Audio S1

Supplementary Information Audio S2

## Figures and Tables

**Table 1:** Characteristics of the horses used in the experiment; breed, sex (F = female; G = gelding), age (in 2013), body weight, as well as the number of whinnies that were analyzed for each horse.

Farm	Horse	Breed	Sex	Age (years)	Weight (kg)*	Number of whinnies
1	1	Swiss Pony	G	20	338	4
	2	English Thoroughbred	G	31	416	21
	3	Swiss Halfbred	F	23	569	35
	4	Swiss Halfbred	G	7	502	22
						
2	1	Swiss Halfbred	F	16	498	8
	2	Swiss Halfbred	F	15	594	7
	3	Swiss Halfbred	F	7	536	2
	4	Irish Sport Horse	F	23	526	2
						
3	1	Akhal-Teke	F	21	404	9
	2	Dartmoor Pony	F	9	267	12
	3	Camargue Horse	F	14	358	18
	4	Quarter Horse	F	12	499	24
						
4	1	French Saddlebred	G	23	593	29
	2	Welsh Pony	G	12	477	25
	3	Swiss Halfbred	F	7	498	5
						
5	1	Swiss Halfbred	F	10	525	0
	2	Swiss Halfbred	G	10	508	10
	3	English Thoroughbred	F	22	516	0
	4	Swiss Halfbred	G	19	403	32
	5	Comtois Horse	G	6	560	2

*Horse body weight was estimated following^64^.

**Table 2:** Abbreviations for the vocal parameters.

Abbreviation	Parameter
Dur (s)	Duration of the whinny
DurIntro (s)	Duration of the introduction part
G0start (Hz)	Frequency value of G0 at the start of the whinny
G0max (Hz)	Maximum G0 frequency value across the whinny
G0mean (Hz)	Mean G0 frequency value across the whinny
F0start (Hz)	Frequency value of F0 at the start of the climax part
F0max (Hz)	Maximum F0 frequency value across the whinny
F0mean (Hz)	Mean F0 frequency value across the whinny
TimeF0max (%)	Percentage of the total whinny duration when F0 is maximum
AmpVar (dB/s)	Cumulative variation in amplitude divided by the total whinny duration
AMrate (s-1)	Number of complete cycles of amplitude modulation per second
AMextent (dB)	Mean peak-to-peak variation of each amplitude modulation
Q25% (Hz)	Frequency value at the upper limit of the first quartiles of energy
Q50% (Hz)	Frequency value at the upper limit of the second quartiles of energy
Q75% (Hz)	Frequency value at the upper limit of the third quartiles of energy
F1mean (Hz)	Mean frequency value of the first putative formant
F2mean (Hz)	Mean frequency value of the second putative formant
F3mean (Hz)	Mean frequency value of the third putative formant
F4mean (Hz)	Mean frequency value of the fourth putative formant

**Table 3:** Effect of emotional arousal level and valence on vocal parameters. Residuals of the linear mixed-effects models controlled for sex, age and body weight of the horses, order of the situations (All or Companion tests first), day of experiment, individual and farm identity (mean ± SD; raw values are listed in Table S1), along with statistical results (likelihood-ratio tests: *χ^2^* values, sample size (*n*) and *p* values). The direction is indicated for the significant (*p* ≤ 0.05) and marginally significant (0.05 < *p* < 0.06) effects (“<” indicates an increase with emotional arousal levels or from negative to positive valence, whereas “>” indicates a decrease; NC indicates that the effect was not consistent, i.e. increase followed by decrease or vice-versa). Significant results are shown in bold (*** indicates *p* < 0.0001). See Table 2 for abbreviations of the parameters.

	Arousal									Valence						
	0		1		2					Negative		Positive				
Parameter	Mean	SD	Mean	SD	Mean	SD	*χ^2^_1_ (n)*	***p***		Mean	SD	Mean	SD	*χ^2^_1_ (n)*	***p***	
**Dur**	**−0.15**	0.60	**−0.03**	0.49	**0.06**	0.42	8.81 (267)	**0.003**	**<**	**0.07**	0.42	**−0.22**	0.56	24.03 (267)	***	**>**
DurIntro	0.01	0.22	**−**0.03	0.17	0.02	0.16	1.22 (267)	0.27		0.01	0.16	**−**0.03	0.20	3.43 (267)	0.064	
**G0start**	**−84.69**	239.29	**−24.21**	237.38	**40.03**	259.79	12.22 (260)	**0.0005**	**<**	**32.69**	235.71	**−108.95**	275.42	19.41 (260)	***	**>**
**G0max**	**−35.73**	156.73	**−7.25**	148.83	**14.70**	147.73	5.09 (260)	**0.024**	**<**	**12.59**	144.13	**−41.98**	161.33	8.00 (260)	**0.005**	**>**
**G0mean**	**−44.03**	137.21	**4.92**	144.18	**7.89**	121.30	3.90 (260)	**0.048**	**<**	**13.33**	127.37	**−44.44**	141.90	11.31 (260)	**0.0008**	**>**
**F0start**	**−17.03**	92.51	**16.74**	109.66	**17.72**	93.99	9.00 (267)	**0.003**	**<**	6.12	98.22	**−**18.62	108.47	3.59 (267)	0.058	>
**F0max**	**−12.08**	71.03	**−9.42**	70.05	**10.65**	80.58	6.66 (267)	**0.010**	**<**	**4.88**	76.22	**−14.85**	73.62	4.14 (267)	**0.042**	**>**
**F0mean**	**−6.98**	59.23	**−10.01**	62.23	**9.74**	74.80	5.42 (267)	**0.020**	**NC**	2.15	71.74	**−**6.55	58.63	0.95 (267)	0.33	
TimeF0max	**−**2.48	15.13	0.28	14.54	0.45	13.31	1.17 (267)	0.28		0.53	13.88	**−**1.61	14.41	1.43 (267)	0.23	
**AmpVar**	**2.19**	9.55	**0.78**	9.60	**−1.20**	7.40	7.57 (267)	**0.006**	**>**	**−0.70**	8.17	**2.12**	9.73	6.57 (267)	**0.010**	**<**
AMrate	**−**0.19	1.92	0.00	1.61	0.05	1.71	0.84 (267)	0.36		0.10	1.64	**−**0.31	1.84	3.63 (267)	0.057	>
**AMextent**	**0.08**	0.32	**0.02**	0.34	**−0.04**	0.31	5.71 (267)	**0.017**	**>**	**−0.03**	0.33	**0.08**	0.31	7.69 (267)	**0.006**	**<**
**Q25%**	**−63.79**	241.79	**−31.83**	287.20	**42.15**	266.57	8.54 (267)	**0.003**	**<**	**18.40**	266.77	**−56.04**	289.64	4.57 (267)	**0.033**	**>**
**Q50%**	**−89.50**	273.94	**−25.36**	268.96	**43.99**	263.68	11.71 (267)	**0.0006**	**<**	**17.35**	251.74	**−52.85**	316.34	4.19 (267)	**0.041**	**>**
**Q75%**	**−0.07**	0.16	**−0.03**	0.17	**0.04**	0.15	26.65 (267)	***	**<**	**0.02**	0.15	**−0.06**	0.19	13.31 (267)	**0.0003**	**>**
F1mean	3.20	36.30	**−**0.60	28.19	**−**0.83	34.43	0.15 (97)	0.70		0.48	32.34	**−**0.94	29.53	0.05 (97)	0.83	
F2mean	**−**17.36	41.94	15.20	47.43	**−**9.45	41.67	0.56 (97)	0.45		**−**0.81	44.98	2.06	48.85	0.13 (97)	0.72	
F3mean	4.67	32.04	0.63	40.99	**−**1.87	35.00	0.31 (131)	0.57		**−**0.50	37.39	1.54	36.95	0.08 (131)	0.78	
F4mean	**−**1.33	36.55	**−**8.42	39.32	13.22	44.78	3.75 (106)	0.053	**NC**	1.70	43.51	**−**4.31	36.93	0.55 (106)	0.46	

**Table 4:** Results of the model comparisons for vocal parameters affected by both emotional arousal and valence. The fit of the models is assessed by Akaike's information criterion adjusted for small sample sizes (AIC_C_). The best model (i.e. arousal or valence; model that best explains the variation in each parameter value) for a given parameter is the model with the lowest AIC_C_ and is indicated in bold. ΔAIC_C_ gives the difference in AIC_C_ between each model and the best model. Akaike's weight (*ωi*) assesses the relative support that a given model has from the data, compared to the other candidate model. See Table 2 for abbreviations of the parameters.

Parameter	Arousal/Valence	AIC_C_	ΔAIC_C_	*ωi*
**Dur**	A	420.35	15.23	0.00
	**V**	**405.13**	**0.00**	**1.00**
**G0start**	A	3696.94	7.19	0.03
	**V**	**3689.75**	**0.00**	**0.97**
**G0max**	A	3444.95	2.91	0.19
	**V**	**3442.04**	**0.00**	**0.81**
**G0mean**	A	3378.43	7.41	0.02
	**V**	**3371.02**	**0.00**	**0.98**
**F0max**	**A**	**3159.55**	**0.00**	**0.78**
	V	3162.07	2.51	0.22
**AmpVar**	**A**	**1950.92**	**0.00**	**0.62**
	V	1951.92	1.00	0.38
**AMextent**	A	204.78	1.98	0.27
	**V**	**202.80**	**0.00**	**0.73**
**Q25%**	**A**	**3831.99**	**0.00**	**0.88**
	V	3835.96	3.97	0.12
**Q50%**	**A**	**3819.71**	**0.00**	**0.98**
	V	3827.23	7.52	0.02
**Q75%**	**A**	**−145.33**	**0.00**	**1.00**
	V	**−**131.99	13.34	0.00
