# Erratum: Segregation of information about emotional arousal and valence in horse whinnies

**DOI:** 10.1038/srep46956

**Published:** 2018-03-19

**Authors:** Elodie F. Briefer, Anne-Laure Maigrot, Roi Mandel, Sabrina Briefer Freymond, Iris Bachmann, Edna Hillmann

Scientific Reports 5: Article number: 9989; 10.1038/srep09989 published online: 04 21 2015; updated: 03 19 2018.

The original version of this Article contained a typographical error in the volume number ‘5’ was incorrectly given as ‘4’. This error has now been corrected in the PDF and HTML versions of the Article.

