# Supplementary material for: Segregation of information about emotional arousal and valence in horse whinnies
Source: Sci Rep. 2015 Apr 21;4:9989. doi: 10.1038/srep09989 (PMC4404681; doi:10.1038/srep09989)
Supplement: Supplementary Information [file srep09989-s1.pdf]

Segregation of information about emotional arousal and valence in horse whinnies

Elodie F. Briefer[1], Anne-Laure Maigrot[1,2*], Roi Mandel[1,3*], Sabrina Briefer Freymond[2], Iris Bachmann[2], Edna Hillmann[1]

[1] *ETH Zürich, Institute of Agricultural Sciences, Universitätstrasse 2, 8092 Zürich, Switzerland*

[2] *Agroscope - Swiss National Stud Farm, Les Longs Prés, P.O. Box 191, 1580 Avenches, Switzerland*

[3] *Koret School of Veterinary Medicine, Robert H. Smith Faculty of Agriculture, Food and Environment, the Hebrew University, Rehovot 76100, Israel*

** Equal contributing authors*

Corresponding author:

Elodie F. Briefer; Email: elodie.briefer@usys.ethz.ch; Tel: +41 (0)44 632 84 55; Address: Institute of Agricultural Sciences, ETH Zürich, Universitätstrasse 2, 8092 Zürich, Switzerland

Supplementary Methods: Acoustic analysis

In this section, we provide a detailed description of the acoustic analysis. The source-related acoustic features, filter-related acoustic features, and intensity features that we measured (19 parameters) are detailed below (Praat commands are indicated in brackets). We extracted all vocal parameters using a custom built program in Praat, which batch processed the analyses and the exporting of output data[1].

Source-related vocal parameters were measured by extracting the fundamental frequency contour of each whinny. Following our analyses, we discovered that all whinnies are composed of two fundamental frequencies that are not harmonically related, "F0" (range = 52-1050 Hz) and "G0" (range = 493-3012 Hz), suggesting biphonation (Figure 2 and 3; Audio S1). After ruling out alterative explanations to biphonation, we analyzed F0 by low-pass filtering whinnies at 200 Hz above F0 (250-1250 Hz). We analyzed G0 by high-pass filtering them above F0 (450-1100 Hz)[2]. Then, we extracted F0 and G0 contours using a cross-correlation method ([Sound: To Pitch (cc) command], F0: time step = 0.01 s, pitch floor = 50 Hz, pitch ceiling = 450-1100 Hz; G0: time step = 0.01 s, pitch floor = 50 Hz, pitch ceiling = 1300-3100 Hz). Using this method, we were usually able to follow G0 in the introduction part, and both F0 and G0 throughout most of the climax, after inspection of the detected contour in Praat [Inspect Pitch object command]. Octave jumps were rectified, if necessary, during inspection of the pitch contour detected by the program. From F0 and G0 contour, we extracted 7 source-related parameters, listed in Table 2 (G0max-TimeF0max), which could be measured in most whinnies.

Filter-related (formants) vocal parameters were measured by extracting the contour of the first four putative formants of the end part of whinnies, which is more noisy and low-pitched than the introduction and climax part (Figure 2). To this aim, we used Linear Predictive Coding analysis (LPC; [Sound: To Formant (burg) command], time step = 0.01 s, maximum number of formants = 5, maximum formant = 1600-2500 Hz, window length = 0.1 s), after assuming an independent source and filter, an

approximately straight uniform vocal tract, and a 6 dB/octave falling spectral slope[3]. The maximum number of putative formants had been determined beforehand as follows. We predicted the expected frequency of each putative formant (F_i) from the following equation, which approximates the vocal tract as a straight uniform tube closed at one end (glottis) and open at the other end (mouth): $F_i = (2_i\text{-}1)c/4VTL$, where c is the speed of sound in air (approximated as 350 m/s in a mammal's vocal tract), and VTL is the length of the vocal tract[1]. The VTL size was estimated by measuring head lengths of each horse (from the tip of the muzzle to the middle of the posterior part of the mandible[4]). The range of head lengths we obtained (35-53 cm) predicts that the first fifth putative formants should be situated below 1486-2250 Hz. We compared the outputs of the LPC analysis with visual inspections of spectrograms to check if Praat accurately tracked the putative formants. Spurious values were deleted and we corrected for octave jumps when necessary. We included in our analyses the mean (F1-4mean) values of the first four putative formants. Additionally, we included in our analyses the frequency values at the upper limit of the first (Q25%), second (Q50%) and third (Q75%) quartiles of energy, measured on a linear amplitude spectrum applied to the whole whinny. These values describe the relative energy distribution in the spectrum.

We measured intensity characteristics by extracting the intensity contour of each whinny [Sound: To Intensity command]. We then included in our analyses the mean variation per second (AmpVar), calculated as the cumulative variation in amplitude divided by the total whinny duration; the number of complete cycles of amplitude modulation per second (AMrate); and the mean peak-to-peak variation of each amplitude modulation (AMextent)[5]. We also included in our analyses the total duration of each whinny (Dur) and the duration of the introduction part (DurIntro). Duration parameters were measured directly on the spectrogram (Figure 2).

G0 and formant-related parameters could not be measured in all the whinnies; G0-related parameters could be measured in 260/267 whinnies (18 horses in total: 93% of the whinnies of each horse on average; range = 50% to 100%). Formants were visible and measured in the end part only, which is more noisy and low-pitched than the

introduction and climax part (Figure 2). Because the end part was not always present in the whinnies that we recorded (Figure 2b), on average, formant values could be measured in 53% of the whinnies of each horse (range = 0% (F1mean for one horse and F4mean for another horse) to 100% (for 3 horses) of the whinnies). All the other parameters could be measured in 100% of the whinnies (see sample sizes in Table 3).

References

1. Reby, D. & McComb, K. Anatomical constraints generate honesty: acoustic cues to age and weight in the roars of red deer stags. *Anim. Behav.* **65,** 519–530 (2003).
2. Volodina, E. V., Volodin, I. A., Isaeva, I. V. & Unck, C. Biphonation may function to enhance individual recognition in the dhole, *Cuon alpinus*. *Ethology* **112,** 815–825 (2006).
3. Boersma, P. [Acoustic analysis] *Research Methods in Linguistics* [Podesva, R. & Sharma, D. (eds.)] [375–396] (Cambridge University Press, Cambridge, UK, 2014).
4. Briefer, E. & McElligott, A. G. Mutual mother–offspring vocal recognition in an ungulate hider species (*Capra hircus*). *Anim. Cogn.* **14,** 585–598 (2011).
5. Charlton, B. D., Zhihe, Z. & Snyder, R. J. Vocal cues to identity and relatedness in giant pandas (*Ailuropoda melanoleuca*). *J. Acoust. Soc. Am.* **126,** 2721–2732 (2009).

Supplementary Results: Physiological and behavioral parameters

To verify further the emotions triggered by our situations, we measured several physiological and behavioral parameters. We measured physiological parameters that are involved in the fight-or-flight stress response (sympathomedullary pathway) and commonly used to assess emotional arousal[6,7]; heart-rate variability (root mean square of successive inter-pulse interval difference, "RMSSD"), respiration rate ("RespRate") and skin temperature ("SkinTemp"; see Table S4 for abbreviations and definitions). Similarly, we scored behavioral parameters that could potentially be affected by emotions[8,9]; locomotion, back and forth returns against the fence, head and ear position, number of whinnies (longest and loudest horse vocalization, mainly aimed at maintaining contact with conspecifics) and nickers (low/medium amplitude vocalization produced with the mouth closed, usually produced before feeding), looking forward, scratching, tail switching and chewing (see Table S4 for abbreviations and definitions[10–12]).

Our results for the physiological parameters revealed that RespRate and SkinTemp were influenced by emotional arousal. RespRate increased with arousal levels, while SkinTemp was not affected in a consistent way (level 0 < 1 > 2). SkinTemp was also influenced by emotional valence (Table S5, and Table S6 for raw values), but, once again, did not change in a consistent way (level - > 0 < +). The other relationships were not significant (Table S5). Model selection using Akaike's information criterion adjusted for small sample size (AIC_C) revealed that the variation in SkinTemp was better explained by valence than arousal (Table S7). However, the ΔAIC_C was very small (0.16), indicating that the models with valence and arousal both had support and were competitive. The model with valence had only 52% chance of being the best model. To summarize, RespRate was a reliable indicator of arousal, as it was changing consistently with arousal levels and was not influenced by valence. There were no clear physiological indicators of valence.

Our behavioral analyses revealed four parameters that were affected by emotional arousal (Locomotion, Returns, HeadMov and Chewing; Table S5, and Table S6 for raw

data). Locomotion, HeadMov and Returns increased with arousal levels, while Chewing decreased. Five parameters were affected by valence (Returns, HeadHigh, HeadMov, NickerRate and Chewing; Table S5, and Table S6 for raw data). HeadHigh decreased and Chewing increased from negative to positive valence. Returns, HeadMov and Nicker changed with valence, but not in a consistent way (Returns, HeadMov and NickerRate: level - > 0 < +). The other parameters were neither affected by arousal nor by valence (Table S5). A model selection procedure based on AIC_C revealed that the variation in Returns was better explained by arousal than valence (Table S7). Conversely, the variation in HeadMov and Chewing was better explained by valence than arousal levels. For Returns and HeadMov, the ΔAIC_C was lower than 2, indicating that the models including arousal and valence were in fact competitive. The model including arousal had 68% chance to be the best model for Return, while the model including valence had 71% chance to be the best model for HeadMov. To summarize, Locomotion was a reliable indicator of emotional arousal, because it was changing consistently with arousal levels and was not influenced by valence. By contrast, HeadHigh and Chewing were good indicators of valence, as they were changing consistently from negative to positive valence and were more affected by valence than arousal levels ($\Delta AIC_C > 9$).

The arousal levels of our emotional situations, based on heart rate values, were confirmed by respiration rate and the time spent moving. During higher arousal situations, horses had higher respiration rates and moved more (Locomotion; walk, trot and canter). The fight-or-flight stress response, triggered by emotional arousal, prepares the animal to react to a stressor by increasing energy supplies for the brain, heart and skeletal muscles[13]. Therefore, this process commonly results in an increase in respiration rate and locomotion[14,15]. Additionally, the valence attributed to our situations was accompanied by changes in the time spent with the head high and the time spent chewing (i.e. moving the lower jaw up and down in a chewing motion without the presence of food). Horses were spending more time with the head above the shoulder in negative than positive situations, which could indicate anxiety/panic[16]. The function of chewing behavior, however, is not clear. It is debated whether it originates from

“snapping”, which is a teeth clapping behavior performed by foals when being approached by adults, or by adults being approached by dominant individuals[11]. Boyd[17] suggested that snapping could serve to calm the submissive individual. The increased time spent chewing during our positive situations, as compared to negative ones, could indicate positive emotions in horses triggered by the sight of their group mate(s) coming back to the stable, following the high-arousal negative emotion triggered by group mate(s) leaving. Therefore, the arousal and valence of our situations were reflected by physiological and behavioral changes in the horses, validating different underlying emotions.

References

6. von Borell, E. *et al.* Heart rate variability as a measure of autonomic regulation of cardiac activity for assessing stress and welfare in farm animals - A review. *Physiol. Behav.* **92,** 293–316 (2007).
7. Reefmann, N., Bütikofer Kaszàs, F., Wechsler, B. & Gygax, L. Physiological expression of emotional reactions in sheep. *Physiol. Behav.* **98,** 235–241 (2009).
8. Reefmann, N., Bütikofer Kaszàs, F., Wechsler, B. & Gygax, L. Ear and tail postures as indicators of emotional valence in sheep. *Appl. Anim. Behav. Sci.* **118,** 199–207 (2009).
9. Imfeld-Mueller, S., Van Wezemael, L., Stauffacher, M., Gygax, L. & Hillmann, E. Do pigs distinguish between situations of different emotional valences during anticipation? *Appl. Anim. Behav. Sci.* **131,** 86–93 (2011).
10. McDonnell, S. M. & Haviland, J. C. S. Agonistic ethogram of the equid bachelor band. *Appl. Anim. Behav. Sci.* **43,** 147–188 (1995).
11. Krueger, K. Behaviour of horses in the ‘round pen technique’. *Appl. Anim. Behav. Sci.* **104,** 162–170 (2007).
12. Yeon, S. C. Acoustic communication in the domestic horse (*Equus caballus*). *J. Vet. Behav.* **7,** 179–185 (2012).
13. Cannon, W. B. *Bodily Changes in Pain, Hunger, Fear and Rage*. (1963).
14. Forkman, B., Boissy, A., Meunier-Salaün, M.-C., Canali, E. & Jones, R. B. A critical review of fear tests used on cattle, pigs, sheep, poultry and horses. *Physiol. Behav.* **92,** 340–374 (2007).
15. Young, T., Creighton, E., Smith, T. & Hosie, C. A novel scale of behavioural indicators of stress for use with domestic horses. *Appl. Anim. Behav. Sci.* **140,** 33–43 (2012).
16. Panksepp, J. The basic emotional circuits of mammalian brains: Do animals have affective lives? *Neurosci. Biobehav. R.* **35,** 1791–1804 (2011).
17. Boyd, L. E. The behaviour of Przewalski’s horses and its importance to their management. *Appl. Anim. Behav. Sci.* **29,** 301–318 (1991).

Supplementary Figures

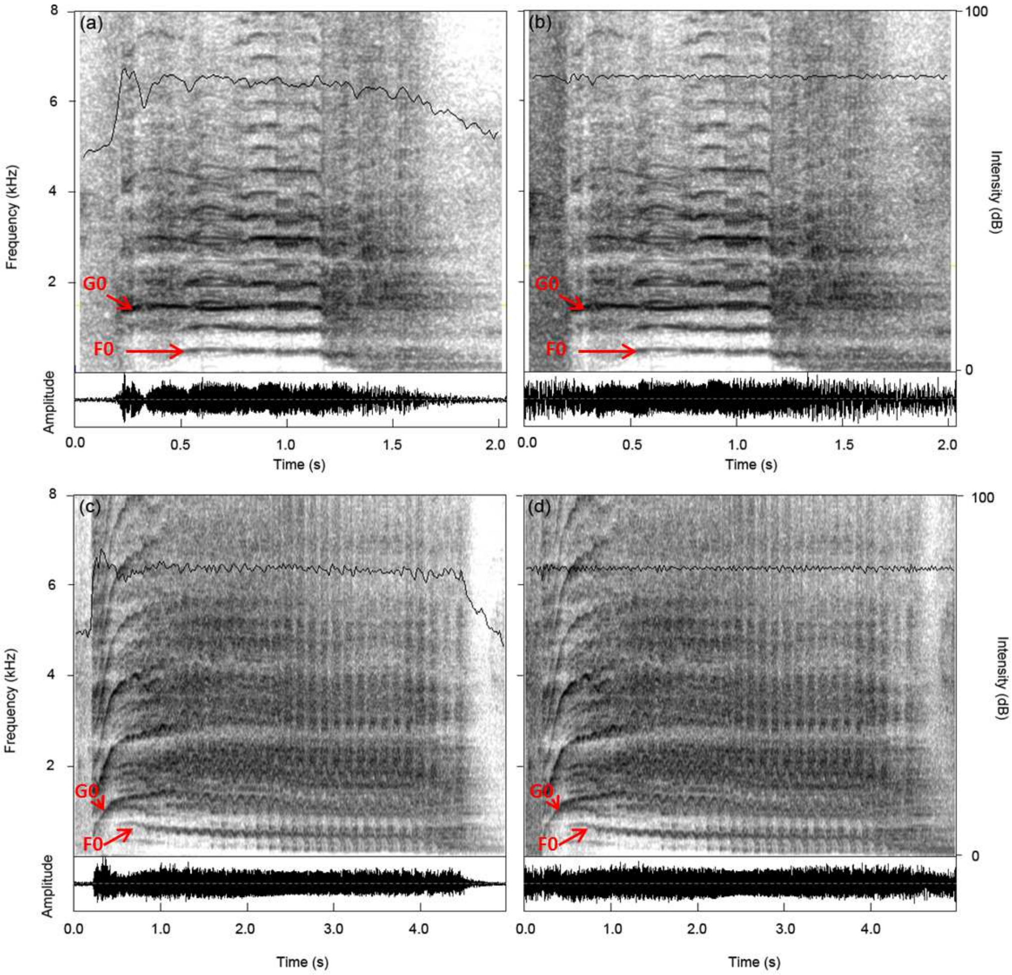

Figure S1 | Effect of the amplitude modulation on the presence of F0 and G0. (a) and (c) spectrograms (above) and oscilliograms (below) of natural whinnies (shown in Figure 1), along with (b) and (d), spectrograms (above) and oscilliograms (below) of the same whinnies artificially modified by removing the amplitude modulation using Praat software. The intensity is shown as a black line on the spectrogram (flat after removing the modulation) and the corresponding scale is displayed on the right (dB). The presence of F0 and G0 is not affected by the modification, suggesting real biphonation.

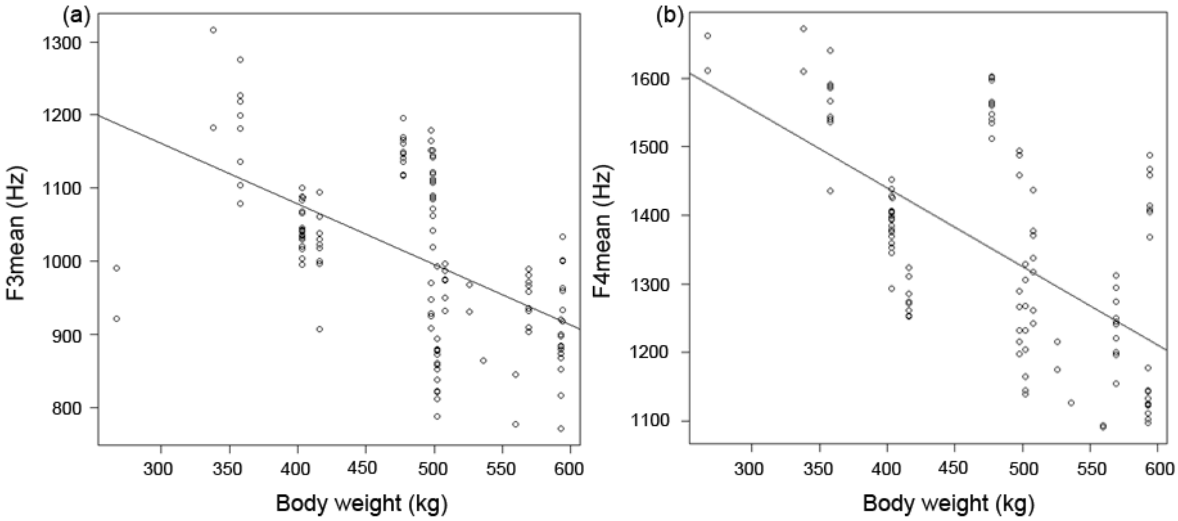

Figure S2 | Relationship between formants and body weight. Horse body weight as a function of (a) F3mean (linear mixed-effects model: χ^2_1 = 6.76, p = 0.009; Table S3) and (b) F4mean (χ^2_1 = 13.49, p = 0.0002; Table S3). Each dot represents a whinny. The best fit is indicated with a solid line.

Supplementary Tables

Table S1 | Raw vocal values. Raw values of the vocal parameters measured for each arousal level and valence (mean ± SD). See Table 2 for abbreviations of the parameters.

	Arousal						Valence			
	0		1		2		-		+	
**Parameters**	**Mean**	**SD**	**Mean**	**SD**	**Mean**	**SD**	**Mean**	**SD**	**Mean**	**SD**
Dur (s)	2.27	0.88	2.16	0.76	2.23	0.70	2.23	0.74	2.14	0.77
DurIntro (s)	0.42	0.58	0.35	0.41	0.55	0.44	0.48	0.45	0.37	0.49
G0start (Hz)	1080.44	231.70	1305.51	365.40	1578.30	408.42	1486.73	399.58	1164.97	364.62
G0max (Hz)	1531.75	237.56	1666.56	351.59	1899.23	381.76	1809.54	373.80	1617.70	368.81
G0mean (Hz)	1309.83	180.32	1471.32	317.00	1657.43	316.69	1588.52	315.17	1392.41	320.46
F0start (Hz)	512.18	161.67	492.65	148.41	547.89	127.77	525.73	128.03	511.08	180.11
F0max (Hz)	571.86	162.38	547.24	114.49	579.64	116.62	565.68	108.87	567.95	160.06
F0mean (Hz)	384.69	87.35	383.81	94.19	415.23	104.44	404.04	100.67	384.55	94.60
TimeF0Max (%)	22.72	20.27	30.44	18.93	35.35	18.65	33.78	18.83	25.84	19.89
AmpVar (dB/s)	35.36	11.94	32.17	10.74	27.92	9.05	29.13	9.53	34.74	11.98
AMrate (s-1)	8.05	2.25	7.76	2.16	7.28	2.43	7.50	2.34	7.74	2.24
AMextent (dB)	4.73	2.04	4.50	2.07	4.13	1.60	4.20	1.79	4.81	2.01
Q25% (Hz)	615.28	318.04	781.89	425.26	899.86	429.59	870.97	433.64	654.62	351.24
Q50% (Hz)	1196.03	314.45	1430.12	395.00	1620.33	351.94	1553.02	357.33	1306.33	432.10
Q75% (Hz)	1813.94	418.22	2127.72	614.53	2392.21	511.64	2297.08	510.62	1966.50	688.04
F1mean (Hz)	237.97	52.59	237.97	40.03	234.21	41.55	240.24	40.77	230.38	45.42
F2mean (Hz)	541.31	72.02	592.55	105.41	536.02	103.59	557.26	99.96	570.20	113.63
F3mean (Hz)	1005.29	85.00	1024.59	119.89	998.28	119.40	1016.08	118.86	992.76	105.99
F4mean (Hz)	1331.85	125.15	1354.17	168.02	1350.17	156.81	1360.95	161.87	1320.28	143.52

Table S2 | Results of the principal component analysis. Factor loadings of the measured vocal parameters on the principal components with eigenvalue > 1 (PC1-PC6) extracted from a principal component analysis including 5 whinnies per individual (*n* = 9 horses). Bold types indicate the heaviest factor loadings (|*r*| >0.50). The six vocal parameters selected as good cues to emotional arousal (F0start, Q50%, Q75%) and valence (Dur, G0start and G0mean) are indicated in bold. Eigenvalues and cumulative explained variances are given at the bottom of the table. See Table 2 for abbreviations of the parameters.

**Parameter**	**PC1**	**PC2**	**PC3**	**PC4**	**PC5**	**PC6**
**Dur**	-0.21	-0.12	-0.47	0.30	-0.22	**0.67**
DurIntro	**-0.56**	0.30	**-0.59**	-0.01	0.13	0.30
**G0start**	-0.23	**-0.74**	0.19	0.25	-0.01	-0.22
G0max	-0.45	**-0.79**	0.17	0.17	0.11	0.15
**G0mean**	-0.35	**-0.83**	0.29	0.12	0.17	-0.05
**F0start**	**0.61**	0.04	-0.28	**0.64**	0.03	-0.06
F0max	**0.71**	-0.02	-0.26	**0.59**	0.06	0.03
F0mean	0.35	0.42	0.10	**0.55**	-0.02	0.17
TimeF0Max	**-0.65**	0.38	0.00	-0.20	0.26	0.06
AmpVar	**0.51**	**-0.60**	-0.09	-0.02	0.21	0.28
AMrate	**0.59**	-0.27	0.26	0.02	**-0.58**	0.16
AMextent	-0.13	-0.24	-0.33	-0.06	**0.79**	0.26
Q25%	**-0.60**	0.44	0.43	0.36	0.07	0.07
**Q50%**	**-0.54**	0.20	**0.58**	**0.50**	0.14	0.07
**Q75%**	**-0.58**	0.10	**0.54**	0.36	-0.10	0.13
F1mean	-0.28	-0.11	0.18	-0.33	**-0.52**	0.49
F2mean	**0.60**	0.14	0.43	0.00	0.26	0.21
F3mean	**0.53**	0.10	**0.55**	-0.42	0.21	0.33
F4mean	**0.69**	0.16	**0.52**	-0.12	0.33	0.13
**Eigenvalue**	**2.22**	**1.76**	**1.63**	**1.45**	**1.31**	**1.13**
**Cum % variance**	26.03	42.28	56.31	67.37	76.36	83.03

Table S3 | Control factors. Effects of the control factors (age, sex, body weight and order of the situations, i.e. All or Companion tests first) on vocal, physiological and behavioral parameters (linear mixed-effects models and generalized linear mixed models, compared with likelihood-ratio tests). Bold font indicates significant ($p \leq 0.05$) or marginally significant ($0.05 < p < 0.06$) results (*** indicates $p < 0.0001$). See Table 2 and S4 for abbreviations of the parameters.

		**Sex**		**Age**		**Weight**		**Order**	
	**Parameter**	X^2_1	***p***	X^2_1	***p***	X^2_1	***p***	X^2_1	***p***
**Vocalizations**	Dur	1.10	0.29	8.78	**0.003**	2.90	0.09	3.73	**0.053**
	DurIntro	2.79	0.09	0.00	0.96	1.32	0.25	1.08	0.30
	G0start	0.75	0.39	0.27	0.61	3.22	0.07	0.52	0.47
	G0max	0.34	0.56	0.05	0.82	3.72	**0.054**	0.34	0.56
	G0mean	0.55	0.46	0.51	0.48	4.01	**0.045**	0.59	0.44
	F0start	0.79	0.38	0.44	0.51	1.00	0.32	0.57	0.45
	F0max	1.59	0.21	0.32	0.57	1.98	0.16	3.51	0.06
	F0mean	7.83	**0.005**	1.45	0.23	4.50	**0.034**	2.10	0.15
	TimeF0Max	0.50	0.48	0.16	0.69	1.26	0.26	0.08	0.78
	AmpVar	7.08	**0.008**	3.55	**0.059**	0.12	0.72	0.78	0.38
	AMeate	0.32	0.57	0.53	0.47	0.60	0.44	0.55	0.46
	AMextent	0.29	0.59	0.01	0.91	0.99	0.32	0.26	0.61
	Q25%	0.76	0.38	0.56	0.46	1.02	0.31	0.12	0.73
	Q50%	0.54	0.46	0.01	0.94	1.88	0.17	0.12	0.73
	Q75%	0.00	0.95	0.08	0.78	1.18	0.28	0.01	0.94
	F1mean	0.82	0.36	1.29	0.26	8.55	**0.003**	0.01	0.90
	F2mean	0.45	0.50	0.08	0.78	10.23	**0.001**	0.03	0.85
	F3mean	0.26	0.61	0.24	0.62	6.76	**0.009**	0.94	0.33
	F4mean	0.55	0.46	0.45	0.50	13.49	**0.0002**	0.60	0.44
**Physiology**	RMSSD	0.05	0.83	0.00	0.99	1.04	0.31	38.17	***
	RespRate	0.60	0.44	0.17	0.68	0.00	0.95	23.44	***
	SkinTemp	1.74	0.19	5.55	**0.019**	10.77	**0.001**	162.03	***
**Behavior**	Locomotion	1.37	0.24	2.62	0.11	3.40	0.065	27.33	***
	Returns	1.28	0.26	0.06	0.81	0.67	0.41	3.35	0.07
	HeadHigh	7.57	**0.006**	5.60	**0.018**	5.09	**0.024**	84.93	***
	HeadMiddle	1.74	0.19	0.85	0.36	6.14	**0.013**	30.24	***
	HeadLow	2.37	0.12	0.29	0.59	0.00	0.95	37.03	***
	HeadMouv	0.90	0.34	0.01	0.90	0.00	0.96	25.59	***
	HeadShaking	1.67	0.20	0.00	1.00	1.03	0.31	12.47	**0.0004**
	EarsBackward	0.13	0.72	0.00	0.95	4.92	**0.027**	48.77	***
	EarsForward	1.40	0.24	2.75	0.10	0.38	0.54	94.20	***
	EarsHorizontal	0.13	0.71	1.21	0.27	0.17	0.68	96.36	***
	EarsAsymetric	2.79	0.10	0.31	0.58	0.34	0.56	67.62	***
	WhinnyRate	1.15	0.28	1.93	0.16	0.00	0.96	11.94	**0.0006**
	NickerRate	2.99	0.08	0.02	0.88	3.53	0.06	7.86	**0.005**
	LookForward	0.42	0.52	1.23	0.27	1.15	0.28	88.56	***
	Scratching	0.00	1.00	0.03	0.87	1.65	0.20	24.60	***
	TailSwishing	0.01	0.92	0.04	0.84	0.01	0.91	13.76	**0.0002**
	Chewing	0.67	0.41	0.57	0.45	0.16	0.69	28.07	***

In addition, the time spent moving (Locomotion) was included as a control factor for the physiological parameters. It had a significant effect on RespRate (LMM: $X^2_1 = 22.88$, $p < 0.0001$;

increase with Locomotion), and SkinTemp (LMM: X^2_1 = 6.68, p = 0.010; increase with Locomotion), but not on RMSSD (LMM: X^2_1 = 1.56, p = 0.21). The outdoor temperature was included as a control factor for SkinTemp and its effect was significant (LMM: X^2_1 = 4.86, p = 0.027; increase with outdoor temperature).

Table S4 | Abbreviations for the physiological and behavioral parameters.

	Abbreviation	Parameter
**Physiology**	HR (beats/min)	Heart rate
	RMSSD (ms)	Root mean square of successive inter-heart-beat interval differences
	RespRate (breaths/s)	Respiration rate
	SkinTemp (°C)	Skin temperature
**Behavior**	Locomotion	Proportion of time spent moving (walk, trot or canter)
	Returns (min^{-1})	Number of way and back movements along the fence or turns inside the stable per minute
	HeadHigh	Proportion of time spent with the eye line above the tip of the shoulder
	HeadMiddle	Proportion of time spent with the eye line at the level of the shoulder tip
	HeadLow	Proportion of time spent with the eye line below the shoulder tip
	HeadMov (min^{-1})	Number of rapid head movements per minute
	HeadShaking (min^{-1})	Number of head shakings per minute
	EarsBackward	Proportion of time spent with the tip of the ears orientated backward
	EarsForward	Proportion of time spent with the tip of the ears orientated forward
	EarsHorizontal	Proportion of time spent with the ears horizontal (perpendicular to the headrump axis)
	EarsAsymetric	Proportion of time spent with the ears asymmetrical (different positions for the right and left ears)
	WhinnyRate (min^{-1})	Number of whinnies per minute
	NickerRate (min^{-1})	Number of nickers per minute
	LookForward	Proportion of time spent looking away and still, with ears orientated forward
	Scratching	Proportion of time spent scratching own body against the fence or using teeth
	TailSwishing (min^{-1})	Number of times the horse whips its tail per minute
	Chewing	Proportion of time spent chewing (i.e. moving the lower jaw up and down in a chewing motion). This behavior is performed without the presence of food in the mouth.

Table S5 | Effect of emotional arousal level and valence on physiological and behavioral parameters. Residuals of the linear mixed-effects models or generalized linear mixed models controlled for Locomotion (physiological parameters only), sex, age and weight of the horses, outdoor temperature (SkinTemp only), order of the situations (All or Companion tests first), day of experiment, individual and farm identity (mean ± SD; raw values are listed in Table S6), along with statistical results (likelihood-ratio tests*:* χ^2 values, sample size (*n*) and *p* values). The direction of the significant effects is indicated ("<" indicates an increase with arousal levels or from negative to positive valence, whereas ">" indicates a decrease; NC indicates that the effect was not consistent, i.e. increase followed by decrease or vice-versa). Significant results are shown in bold (*** indicates $p < 0.0001$). See Table S4 for abbreviations of the parameters.

Dimension		0		1		2				
	**Parameter**	**Mean**	SD	**Mean**	SD	**Mean**	SD	χ^2_1 *(n)*	***p***	
**Arousal**	RMSSD	0.09	0.55	-0.03	0.57	-0.06	0.55	2.16 (214)	0.14	
	**RespRate**	**-0.12**	0.32	**0.06**	0.31	**0.06**	0.29	6.26 (214)	**0.012**	**<**
	**SkinTemp**	**-0.18**	1.89	**0.21**	1.47	**-0.17**	1.30	4.06 (214)	**0.044**	**NC**
	**Locomotion**	**-0.28**	1.02	**0.12**	1.05	**0.22**	1.01	5.00 (225)	**0.025**	**<**
	**Returns**	**-0.40**	0.43	**-0.04**	0.77	**0.07**	0.85	11.05 (225)	**0.0009**	**<**
	HeadHigh	-0.11	1.91	-0.17	1.78	0.51	1.57	3.04 (225)	0.08	
	HeadMiddle	0.11	1.13	-0.11	1.05	-0.16	1.06	0.06 (225)	0.80	
	HeadLow	0.01	1.07	-0.30	0.89	-0.10	0.99	0.42 (225)	0.52	
	**HeadMov**	**-0.41**	1.07	**0.13**	1.07	**0.45**	0.93	9.03 (225)	**0.003**	**<**
	HeadShaking	-0.18	0.76	-0.17	0.75	-0.13	0.77	0.16 (225)	0.69	
	EarsBackward	0.11	1.12	-0.28	0.84	-0.38	0.66	1.35 (225)	0.25	
	EarsForward	-0.35	1.24	0.16	0.99	0.19	0.79	1.31 (225)	0.25	
	EarsHorizontal	0.23	1.38	-0.16	1.14	-0.01	0.64	0.40 (225)	0.53	
	EarsAsymetric	-0.06	0.85	0.04	0.83	0.00	0.67	1.42 (225)	0.23	
	Whinnies	-0.29	0.87	-0.09	0.90	0.12	0.96	2.10 (225)	0.15	
	Nicker	-0.24	0.80	-0.09	0.94	-0.29	0.76	0.74 (225)	0.39	
	LookForward	-0.40	1.74	0.32	1.54	-0.07	1.41	0.18 (225)	0.67	
	Scatching	-0.06	0.68	-0.17	0.49	-0.22	0.34	0.13 (225)	0.72	
	TailSwishing	-0.16	0.55	-0.13	0.63	-0.20	0.44	0.01 (225)	0.92	
	**Chewing**	**0.21**	1.17	**-0.25**	0.90	**-0.54**	0.56	23.78 (225)	***	**>**
		**Negative**		**Neutral**		**Positive**				
	**Parameter**	**Mean**	SD	**Mean**	SD	**Mean**	SD	χ^2_1 *(n)*	***p***	
**Valence**	RMSSD	-0.03	0.61	0.20	0.55	-0.01	0.50	0.24 (214)	0.63	
	RespRate	0.02	0.28	-0.28	0.28	0.03	0.33	0.00 (214)	0.99	
	**SkinTemp**	**-0.04**	1.39	**-1.82**	2.31	**0.37**	1.39	4.22 (214)	**0.040**	**NC**
	Locomotion	0.16	1.02	-0.55	1.02	-0.04	1.05	2.05 (225)	0.15	
	**Returns**	**0.05**	0.84	**-0.48**	0.32	**-0.25**	0.60	9.52 (225)	**0.002**	**NC**
	**HeadHigh**	**0.36**	1.66	**-0.14**	1.94	**-0.33**	1.85	8.28 (225)	**0.004**	**>**
	HeadMiddle	-0.01	1.07	0.72	0.99	-0.23	1.04	2.56 (225)	0.11	
	HeadLow	-0.17	0.96	0.64	1.16	-0.28	0.89	0.84 (225)	0.36	
	**HeadMov**	**0.36**	0.98	**-1.25**	0.30	**-0.09**	1.09	10.79 (225)	**0.001**	**NC**
	HeadShaking	-0.11	0.80	-0.22	0.71	-0.22	0.72	1.28 (225)	0.26	
	EarsBackward	-0.28	0.81	1.09	1.10	-0.29	0.83	0.01 (225)	0.93	
	EarsForward	0.22	0.78	-1.36	1.08	0.03	1.14	1.99 (225)	0.16	
	EarsHorizontal	-0.15	0.85	1.40	1.15	-0.10	1.25	0.14 (225)	0.71	
	EarsAsymetric	0.01	0.77	-0.74	0.74	0.12	0.79	0.95 (225)	0.33	
	WhinnyRate	-0.10	0.88	-0.75	0.32	-0.01	0.97	0.40 (225)	0.53	
	**NickerRate**	**-0.33**	0.70	**-0.55**	0.14	**0.03**	1.01	10.36 (225)	**0.001**	**NC**
	LookForward	-0.04	1.34	-1.79	0.85	0.35	1.74	3.29 (225)	0.07	
	Scratching	0.02	0.13	0.20	0.41	0.04	0.21	1.35 (225)	0.25	
	TailSwishing	-0.18	0.53	-0.02	0.75	-0.15	0.56	0.09 (225)	0.76	
	**Chewing**	**-0.53**	0.58	**-0.07**	1.09	**0.18**	1.14	33.74 (225)	***	**<**

Table S6 | Raw physiological and behavioral values. Raw values of the physiological and behavioral parameters measured for each arousal level and valence (mean ± SD). See Table S4 for abbreviations of the parameters.

	Arousal						Valence					
	0		1		2		Negative		Neutral		Positive	
**Parameter**	**Mean**	**SD**	**Mean**	**SD**	**Mean**	**SD**	**Mean**	**SD**	**Mean**	**SD**	**Mean**	**SD**
HR (beats/min)	43.93	11.58	50.27	16.96	56.32	23.46	53.04	20.90	41.34	7.54	47.34	14.29
RMSSD (ms)	62.58	44.38	57.78	52.52	54.25	36.87	58.47	52.84	67.38	38.51	57.24	41.99
RespRate (breaths/s)	0.41	0.19	0.54	0.28	0.53	0.23	0.51	0.24	0.34	0.16	0.50	0.26
SkinTemp (°C)	29.01	3.70	29.94	3.67	29.70	3.70	29.59	3.72	26.85	2.38	30.06	3.67
Locomotion (%)	13.65	19.99	21.30	23.79	27.34	27.83	25.05	26.06	21.97	0.00	21.37	95.87
Returns (min^{-1})	0.10	0.54	0.71	1.84	1.09	2.37	1.02	2.29	0.00	0.00	0.28	0.96
HeadHigh (%)	51.36	34.48	51.65	30.95	63.94	30.12	60.11	30.47	49.60	36.62	49.19	32.48
HeadMiddle (%)	9.45	18.47	7.18	16.26	4.91	10.98	7.25	15.08	13.65	22.46	6.50	15.68
HeadLow (%)	12.94	28.08	5.37	18.46	3.42	13.50	3.59	12.49	32.75	38.30	6.73	21.88
HeadMouv (min^{-1})	7.35	5.47	10.43	6.14	14.30	7.72	12.57	7.09	3.10	1.91	9.24	5.82
HeadShaking (min^{-1})	0.30	1.09	0.34	1.04	0.60	1.90	0.52	1.56	0.11	0.25	0.30	1.08
EarsBackward (%)	3.85	11.47	1.97	7.08	0.57	1.96	1.64	6.48	10.15	19.33	1.52	5.51
EarsForward (%)	38.85	28.66	48.65	25.01	48.42	23.95	49.54	23.16	19.70	18.16	45.85	28.08
EarsHorizontal (%)	41.15	27.94	32.29	23.39	34.40	18.66	32.00	19.41	62.90	24.04	34.42	25.87
EarsAsymetric (%)	16.34	14.92	17.23	12.98	16.64	11.98	16.94	12.95	7.50	8.29	18.34	13.97
WhinnyRate (min^{-1})	0.50	1.08	0.71	1.26	1.55	2.79	1.02	2.16	0.00	0.00	0.78	1.24
NickerRate (min^{-1})	0.55	1.74	0.61	1.54	0.19	0.59	0.21	0.82	0.00	0.00	0.87	1.94
LookForward (%)	26.04	30.08	35.13	29.75	27.42	27.01	28.79	26.50	3.45	6.79	36.96	31.86
Scratching (%)	0.28	1.30	0.57	4.53	0.06	0.41	0.09	0.72	0.45	1.19	0.62	4.56
TailSwishing (min^{-1})	0.07	0.32	0.13	0.49	0.08	0.38	0.09	0.41	0.06	0.16	0.11	0.45
Chewing (%)	6.70	13.98	2.69	9.07	0.51	2.53	0.96	5.45	1.20	2.24	6.49	13.70

Table S7 | Results of the model comparisons for physiological and behavioral parameters affected by both emotional arousal and valence. The fit of the models is assessed by Akaike's information criterion corrected for small sample sizes (AIC_C). The best model (i.e. arousal or valence; model that best explains the variation in each parameter value) for a given parameter is the model with the lowest AIC_C and is indicated in bold. ΔAIC_C gives the difference in AIC_C between each model and the best model. Akaike's weight (*ωi*) assesses the relative support that a given model has from the data, compared to the other candidate model. See Table S4 for abbreviations of the parameters.

	**Parameter**	**Arousal/ Valence**	**AIC_C**	**ΔAIC_C**	***ωi***
**Physiology**	**SkinTemp**	A	838.56	0.16	0.48
		**V**	**838.40**	**0.00**	**0.52**
**Behavior**	**Returns**	**A**	**172.65**	**0.00**	**0.68**
		V	174.19	1.53	0.32
	**HeadMov**	A	291.96	1.76	0.29
		**V**	**290.20**	**0.00**	**0.71**
	**Chewing**	A	221.04	9.96	0.01
		**V**	**211.08**	**0.00**	**0.99**

Supplementary Audio files

Audio S1 | Horse whinnies. Audio file of the two whinnies shown in Figure 2 (a and b), separated by 2 s of silence. These whinnies are produced by different horses.

Audio S2 | Horse whinnies produced in negative and positive situation, corresponding to Figure 3 (a, b, c and d). The audio file is composed of four whinnies produced by two different horses. The first two whinnies, separated by 2 s of silence, correspond to the first horse (negative and positive; Figure a and b, respectively). The next two whinnies, also separated by 2 s of silence, correspond to the second horse (negative and positive; Figure b and c, respectively). The two pairs of whinnies are separated by 5 s-silence interval.
